# SARS-CoV-2 Antibody Seroprevalence in Wuhan, China, from 23 April to 24 May 2020

**DOI:** 10.1128/mSphere.01062-20

**Published:** 2021-04-21

**Authors:** Huan Han, Junzhu Yi, Gongbo Cheng, Wuhui Jiang, Grzegorz M. Brożek, Yingan Jiang, Chengliang Zhu, Yuchen Xia

**Affiliations:** a Department of Clinical Laboratory, Renmin Hospital of Wuhan University, Wuhan, China; b State Key Laboratory of Virology, Institute of Medical Virology, School of Basic Medical Sciences, Wuhan University, Wuhan, China; c Hubei Province Key Laboratory of Allergy and Immunology, Institute of Medical Virology, School of Basic Medical Sciences, Wuhan University, Wuhan, China; d Guangdong Provincial Engineering Technology Research Center of Environmental and Health Risk Assessment, Department of Occupational and Environmental Health, School of Public Health, Sun Yat-Sen University, Guangzhou, China; e Department of Epidemiology, School of Medicine, Medical University of Silesia, Katowice, Poland; f Department of Infectious Diseases, Renmin Hospital, Wuhan University, Wuhan, China; Mount Sinai School of Medicine

**Keywords:** COVID-19, IgG, IgM, Wuhan, antibody

## Abstract

The outbreak of coronavirus disease 2019 (COVID-19) was first reported in Wuhan, China, in December 2019. To investigate the prevalence of COVID-19 in Wuhan, we conducted serologic tests on 35,326 individuals from four different communities to estimate cumulative incidence of infection. Our results showed that 1,332 individuals (3.77%) showed positive COVID-19 antibody (either IgM or IgG). Males had a lower positivity rate than females (3.02% versus 4.52%). The antibody positivity rates showed a clear trend of increase according to patients’ ages and varied among different communities. The results indicate that public health interventions may play important roles in the control of COVID-19.

**IMPORTANCE** Coronavirus disease 2019 (COVID-19) was first detected in December 2019 in Wuhan, China. Afterwards, a number of public health interventions were implemented, including lock-down, face mask ordinances, and social distancing. Studies that rely on viral RNA testing of symptomatic patients have shown that these multifaceted interventions contributed to the control of the COVID-19 outbreak in Wuhan and delayed the epidemic’s progression. However, these estimates of confirmed cases may miss large numbers of asymptomatic patients and recovered symptomatic patients who were not tested. To investigate the prevalence of COVID-19 in Wuhan, we conducted serologic tests on 35,326 individuals to estimate the cumulative incidence of infection. The results suggest that public health interventions may play important roles in the control of COVID-19.

## INTRODUCTION

Coronavirus disease 2019 (COVID-19) was first detected in December 2019 in Wuhan, China. Studies suggested that early transmission had occurred among close contacts since the middle of December 2019 (1). After the announcement of human-to-human transmission, which was made on 20 January 2020, numbers of public health interventions were implemented, including a lock-down of Wuhan city and later all of Hubei Province, urban traffic restrictions, social distancing, face mask ordinances, stay-at-home policies, temporary hospitals, and centralized quarantine (2). As of 28 January 2021, there were 50,355 confirmed COVID-19 cases in Wuhan.

A modeling study estimated that the Wuhan travel ban delayed the epidemic progression by 3 to 5 days in mainland China and reduced international case importations by 80% through mid-February (3). These multifaceted interventions were temporally associated with improved control of the COVID-19 outbreak in Wuhan (2). The potential effect of such social distancing interventions on severe acute respiratory syndrome coronavirus 2 (SARS-CoV-2) spread and COVID-19 burden was mitigated in Singapore, China (4). However, the determination of COVID-19 cases in most of those studies relied on the detection of SARS-CoV-2 viral RNA in symptomatic patients. These estimates of confirmed cases may miss large numbers of asymptomatic patients and recovered symptomatic patients who were not tested. To investigate the prevalence of COVID-19 in Wuhan, we conducted serologic tests on 35,326 individuals to estimate the cumulative incidence of infection.

## RESULTS

**FIG 1:**
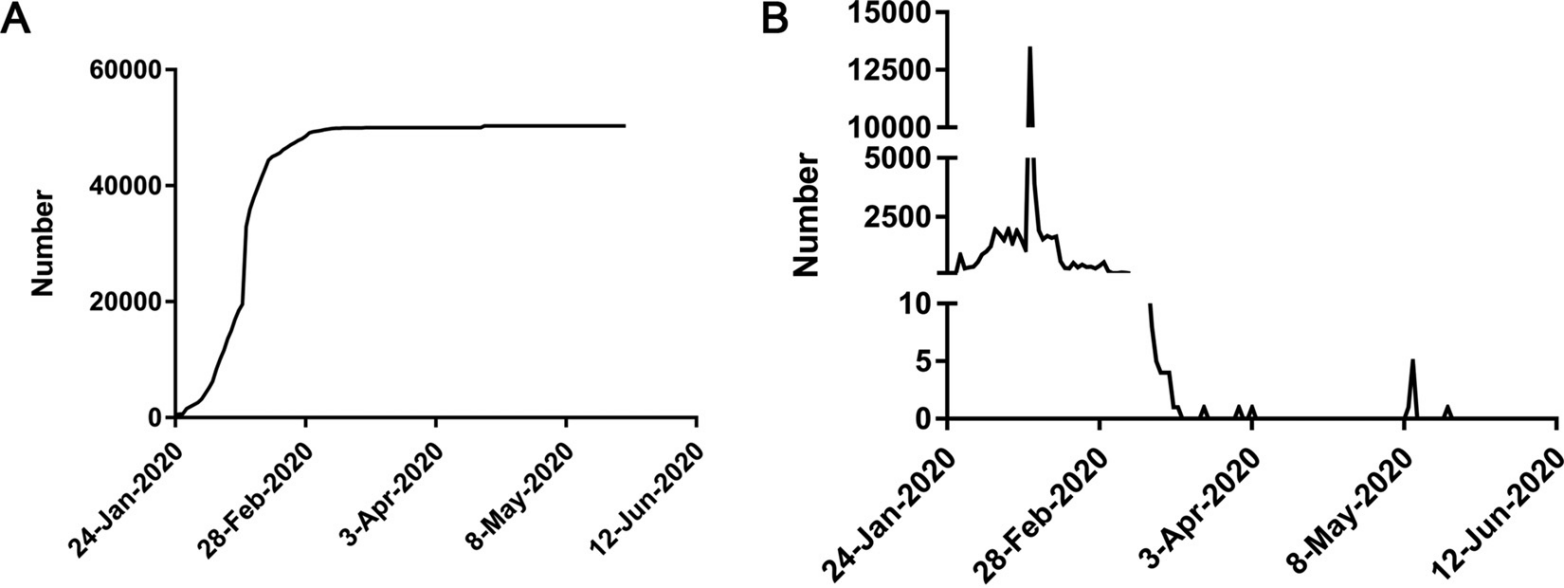
COVID-19 prevalence in Wuhan, China. Numbers of cumulative (A) and incident (B) cases in Wuhan over time.

The first confirmed COVID-19 case of Wuhan was reported on 31 December 2019. The number increased rapidly until 18 February 2020 and flattened afterwards, as shown by the kinetics of both the cumulative COVID-19 case numbers (Fig. 1A) and the incidence of new cases (Fig. 1B). As of 24 May 2020, the total number of confirmed cases of the COVID-19 had increased to 50,340 in Wuhan (Fig. 1A). During our sampling period (from 23 April 2020 to 24 May 2020), only 7 confirmed cases were found (Fig. 1B).

In our serologic tests, the initial screen was conducted with a colloidal-gold-based immunochromatographic assay (Vazyme, Nanjing, China) with 91.54% sensitivity and 97.02% specificity. A second test with a quantitative chemiluminescent immunoassay (YHLO Biotech, Shenzhen, China) was performed to confirm all positive samples. The sensitivity and specificity for IgM are 88.2% and 99.0%, respectively, and those for IgG are 97.8% and 97.9%, respectively. The COVID-19 antibody seroprevalence was reported for the whole study population and different age and gender subgroups. Difference in prevalence between subgroups was examined by the chi-square test. Confidence intervals of prevalence were estimated using a bootstrap procedure.

**FIG 2:**
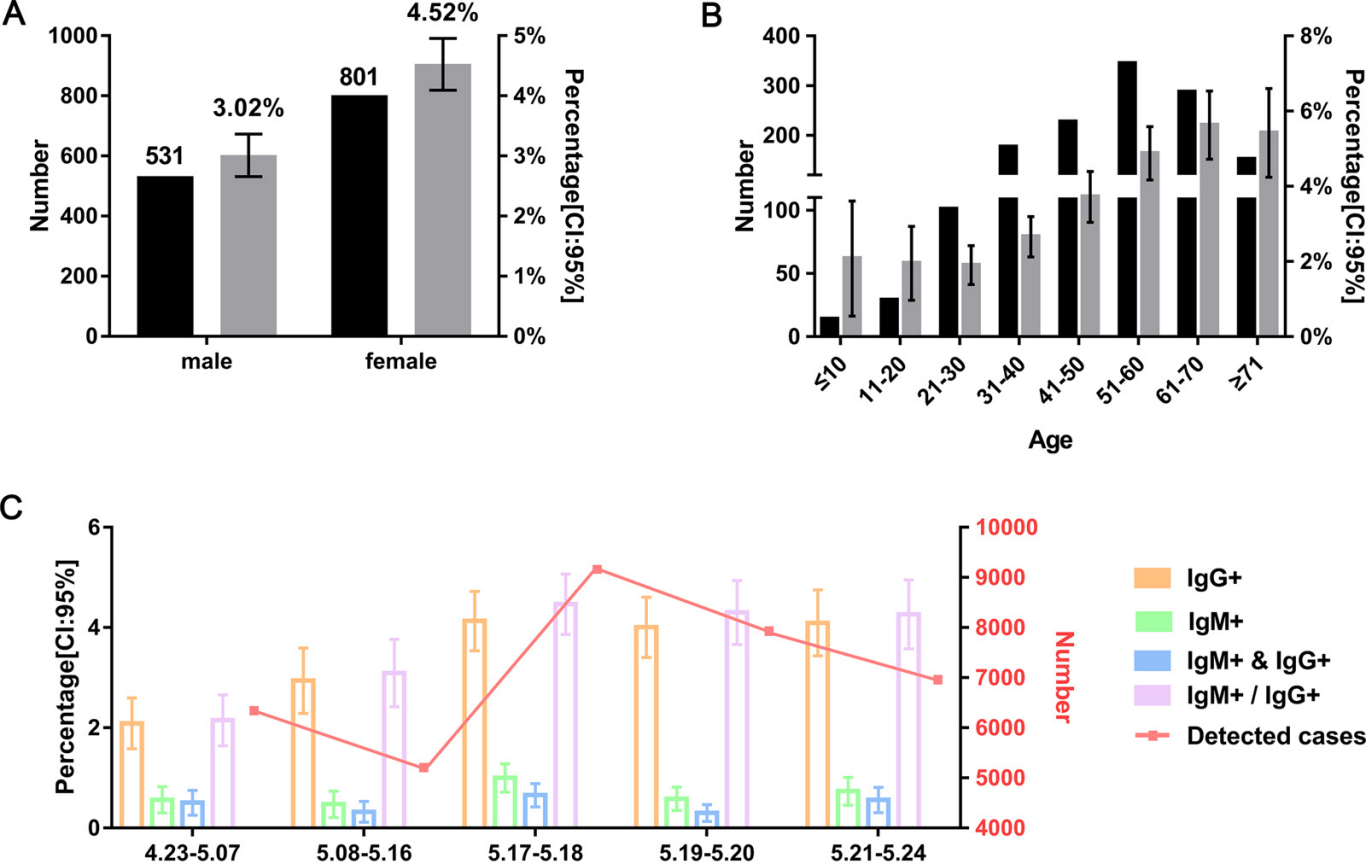
SARS-CoV-2 antibody seroprevalence in Wuhan, China. (A and B) Numbers (black bars) and proportions (gray bars) of COVID-19 antibody-positive (IgG or IgM) individuals divided by gender (A) or age (B). (C) COVID-19 antibody seroprevalence (colored bars) and detected cases (red line) in this study at different periods of time. Error bars represent 95% confidence intervals (95% CI). IgG^+^, positivity for IgG; IgM^+^, positivity for IgM; IgG^+^& IgM^+^, positivity for both IgG and IgM; IgG^+^/IgM^+^, positivity for either IgG or IgM.

Out of 35,326 individuals included, 49.85% were male and 56.23% were 31 to 60 years old (Table 1). As shown in Table 2 and Fig. 2, the overall positivity rate for IgG was 3.55% (bootstrap 95% confidence interval [CI], 3.35%, 3.74%), and that for IgM was 0.70% (bootstrap 95% CI, 0.61%, 0.78%). While 1,332 individuals, which account for 3.77% (bootstrap 95% CI, 3.58%, 3.97%) of the cohort, tested positive for COVID-19 antibody (either IgM or IgG), 168 people (0.48%; bootstrap 95% CI, 0.40%, 0.55%) were positive for both IgG and IgM. Among the 1,332 who tested positive, 531 were males and 801 were females. Interestingly, males had a lower positivity rate than females (3.02%; bootstrap 95% CI, 2.77%, 3.27%, versus 4.52%; bootstrap 95% CI, 4.22%, 4.83% [χ^2^ = 55; *P* < 0.001]) (Fig. 2A). The proportions of subjects positive for IgM or IgG varied by age and showed a clear trend of increase according to the increase of patients’ ages (Fig. 2B), which are in line with previous reports (2). We also compared seropositivity rates at different periods of time. The antibody positivity rate increased from April 23th to May 18th and stabilized afterwards (Fig. 2C).

**TABLE 1:** Basic information about the cohort

Parameter	No. of patients	%
Gender		
Male	17,610	49.85
Female	17,716	50.15

Age (yrs)		
≤10	697	1.97
11−20	1,512	4.28
21–30	5,333	15.10
31–40	6,674	18.89
41–50	6,115	17.31
51–60	7,076	20.03
61–70	5,106	14.45
≥71	2,813	7.96

**TABLE 2:** Numbers and percentages of COVID-19-positive individuals

Parameter	No. (%) of patients with^*a*^:			
	IgG^+^	IgM^+^	IgG^+^ & IgM^+^	IgG^+^/IgM^+^
Total	1,254 (3.55)	246 (0.70)	168 (0.48)	1,332 (3.77)
Male	510 (2.90)	107 (0.61)	86 (0.49)	531 (3.02)
Female	744 (4.20)	139 (0.78)	82 (0.46)	801 (4.52)
Age (yrs):				
≤10	14 (2.01)	1 (0.14)	1 (0.14)	14 (2.01)
11−20	29 (1.92)	3 (0.20)	3 (0.20)	29 (1.92)
21−30	91 (1.71)	14 (0.26)	4 (0.08)	101 (1.89)
31-40	167 (2.50)	27 (0.40)	17 (0.25)	177 (2.65)
41-50	215 (3.52)	39 (0.64)	27 (0.44)	227 (3.71)
51−60	317 (4.48)	80 (1.13)	52 (0.73)	345 (4.88)
61−70	278 (5.44)	48 (0.94)	39 (0.76)	287 (5.62)
≥71	143 (5.08)	34 (1.21)	25 (0.89)	152 (5.40)

a IgG^+^, positivity for IgG; IgM^+^, positivity for IgM; IgG^+^& IgM^+^, positivity for both IgG and IgM; IgG^+^/IgM^+^, positivity for either IgG or IgM.

As a human-to-human transmissive disease, COVID-19 may occur in clusters, so we next investigated whether there was a difference in seroprevalence in the different communities that had been sampled. We investigated 26,274 out of 35,326 individuals who provided detailed home address information and were sampled from May 12th to May 24th. As shown in Table 3, although all four communities had similar IgM positivity rates, the Pengliuyang community had a higher IgG positivity rate than others, suggesting that more virus transmission occurred in Pengliuyang than in the other three communities.

**TABLE 3:** Numbers and percentages of COVID-19-positive individuals in the four communities from 12 May 2020 to 24 May 2020

Parameter	No. (%) of patients	No. (%) of patients with^*a*^:			
		IgG^+^	IgM^+^	IgG^+^ & IgM^+^	IgG^+^/IgM^+^
Total	26,274	1,052 (4.00)	204 (0.78)	133 (0.51)	1,123 (4.27)
Dushuyuan	6,870 (26.15)	272 (3.96)	53 (0.77)	39 (0.57)	286 (4.16)
Huatijie	4,159 (15.83)	140 (3.37)	32 (0.77)	16 (0.38)	156 (3.75)
Pengliuyang	8,718 (33.18)	403 (4.62)	68 (0.78)	49 (0.56)	422 (4.84)
Xichangkou	6,527 (24.84)	237 (3.63)	51 (0.78)	29 (0.44)	259 (3.97)
χ^2^		15.479	0.008	2.771	11.320
*P*		0.001	1.000	0.428	0.010

a IgG^+^, positivity for IgG; IgM^+^, positivity for IgM; IgG^+^& IgM^+^, positivity for both IgG and IgM; IgG^+^/IgM^+^, positivity for either IgG or IgM.

## DISCUSSION

The results of this study provide important data to assess the state of the COVID-19 epidemic in the former epicenter, Wuhan. At the end of the first wave of the pandemic in Wuhan, about 3.77% of people had developed detectable antibodies against SARS-CoV-2. Our results suggest that the number of infections was greater than the number of reported cases, which is likely due to asymptomatic infections. Currently, the widely used symptom-based screening is insufficient by itself to detect a proportion of potentially infectious cases and to control transmission. Although it is a time- and cost-consuming task to test all residents, a “pooled testing” strategy to screen many residents at once could be considered.

In this study, we found that elders had a higher SARS-CoV-2 antibody prevalence. It is known that elderly people have a higher proportion of comorbid conditions, which might facilitate SARS-CoV-2 infection and increase the severity of COVID-19 (2). Previous research indicated that more severe infections result in higher and longer antibody responses (5), so we could not conclude that elders are more susceptible to COVID-19.

Previous studies showed that some patients still have a detectable level of IgM more than 30 days after disease onset (6, 7). Additionally, since asymptomatic patients have no clinical symptoms that can delay timely diagnosis and treatment, they may represent a greater risk of virus transmission than symptomatic patients, posing a major challenge to infection control (8). Our detection of a relatively high proportion of IgM-positive individuals at a time when only 7 cases were confirmed by reverse transcription-PCR (RT-PCR) testing may suggest that asymptomatic virus transmission was continuing, undetected, in these communities. Together, these may be the reasons that we observed a rather high percentage of IgM antibodies.

Duan et al. collected 63,107 healthy persons from 30 provinces in mainland China from 6 March to 3 May 2020, 11,086 of whom were from Wuhan (14). The serology test was performed by using commercial colloidal-gold detection kits, with the recombinant SARS-CoV-2 N proteins as the antigens (1). Liu et al. investigated 35,040 individuals in Wuhan between 27 March and 26 May 2020. This study used one commercial kit and found that the seropositivity prevalence was 3.9% (9). A recent study investigated a total of 17,368 individuals from four different geographic locations and different populations in China, 1,279 of whom were health care workers and their relatives or hotel staff in Wuhan (10). Their study demonstrated that the rates of seropositivity in Wuhan varied between 3.2% and 3.8% in those particular populations. In our studies, we got a similar seropositivity rate of 3.77%. Compared with that of other studies, the overall sample size was relatively large and contained all age groups. In addition, we focused on all residents instead of particular populations. Furthermore, we used two different immunoassays to detect the antibodies, which results in a high specificity and a low rate of false-positive results. These provide important data to assess the state of the COVID-19 epidemic in Wuhan.

Of note, the overall positive rate in Wuhan is lower than what was reported in other regions, like Spain (3.77% versus 5.0% to ∼6.2%) (11) and Geneva, Switzerland (3.77% versus 10.8%) (12). Although the difference between those studies and ours may be due to different ethnicities or detection methods, our results also suggest that public health interventions executed during late January to early March may have played important roles in the control of COVID-19 in Wuhan. Beyond the current phase of the pandemic, it may be necessary to proceed with a cautious approach in reopening businesses in areas of epidemicity to prevent potential future waves of COVID-19.

## MATERIALS AND METHODS

### Sample collection.

We recruited 35,326 residents (17,610 males and 17,716 females greater than 4 years old) from four communities (Dushuyuan, Huatijie, Pengliuyang, and Xichangkou) in Wuhan from 23 April 2020 to 24 May 2020. All participants had not previously tested positive for COVID-19 before providing serum samples for this study. Blood samples were drawn from residents and were immediately separated by centrifugation at 3,000 rpm for 5 min to get serum for SARS-CoV-2 antibody detection. Serologic testing for SARS-CoV-2 IgM and IgG antibodies was performed at Renmin Hospital of Wuhan University.

### SARS-CoV-2 antibody detection.

The 2019-nCoV IgG/IgM detection kit (colloidal-gold based, catalog no. C6603C; Vazyme, Nanjing China) was used for the first-round screen of SARS-CoV-2 antibody, with 91.54% sensitivity and 97.02% specificity reported by the manufacturer. If the results for the IgG and/or IgM antibody were positive, the samples would be tested with the YHLO chemiluminescence IgG immunoassay (CLIA-IgG) and YHLO CLIA IgM kits (catalog no. C86095M; YHLO Biotech, Shenzhen, China) according to the manufacturer’s instructions. The kit contains two SARS-CoV-2 recombinant antigens, nucleoprotein and spike protein. The sensitivity and specificity for IgM are 88.2% and 99.0%, respectively, and for IgG are 97.8% and 97.9%, respectively. Only confirmed cases were counted.

### Statistical analysis.

The COVID-19 antibody seroprevalence was reported for the whole study population and different age and gender subgroups. Difference in prevalence between subgroups was examined by the chi-square test with SPSS software. Confidence intervals of prevalence were estimated using a bootstrap procedure with software R as described previously (13). Due to the lack of standard samples (all COVID-19 patients’ samples during the epidemic have been destroyed due to a biosafety regulation), the estimated prevalence has not been adjusted according to the sensitivity and specificity of the test kits.

### Ethics statement.

The study was approved by the ethics committee of Renmin Hospital of Wuhan University (file no. WDRY2020-K066). Formal consent was obtained from each individual or guardian.
